# Failure of Healthcare Provision for Attention-Deficit/Hyperactivity Disorder in the United Kingdom: A Consensus Statement

**DOI:** 10.3389/fpsyt.2021.649399

**Published:** 2021-03-19

**Authors:** Susan Young, Philip Asherson, Tony Lloyd, Michael Absoud, Muhammad Arif, William Andrew Colley, Samuele Cortese, Sally Cubbin, Nancy Doyle, Susan Dunn Morua, Philip Ferreira-Lay, Gisli Gudjonsson, Valerie Ivens, Christine Jarvis, Alexandra Lewis, Peter Mason, Tamsin Newlove-Delgado, Mark Pitts, Helen Read, Kobus van Rensburg, Bozhena Zoritch, Caroline Skirrow

**Affiliations:** ^1^Psychology Services Limited, London, United Kingdom; ^2^Department of Psychology, Reykjavik University, Reykjavik, Iceland; ^3^ADHD Foundation, Liverpool, United Kingdom; ^4^Social, Genetic and Developmental Psychiatry Centre, Institute of Psychiatry, Psychology & Neuroscience, Kings College London, London, United Kingdom; ^5^South London and Maudsley NHS Foundation Trust, London, United Kingdom; ^6^Department of Children's Neurosciences, Evelina London Children's Hospital, Guy's and St Thomas' NHS Foundation Trust, London, United Kingdom; ^7^King's Health Partners Academic Health Science Centre, London, United Kingdom; ^8^Department of Women and Children's Health, School of Life Course Sciences, Faculty of Life Sciences and Medicine, King's College London, London, United Kingdom; ^9^Leicestershire Partnership NHS Trust, Leicester, United Kingdom; ^10^CLC Consultancy, Perth, United Kingdom; ^11^SWB (Global), Glasgow, United Kingdom; ^12^Centre for Innovation in Mental Health, School of Psychology, Faculty of Environmental and Life Sciences & Clinical and Experimental Sciences (CNS and Psychiatry), Faculty of Medicine, University of Southampton, Southampton, United Kingdom; ^13^Solent NHS Trust, Southampton, United Kingdom; ^14^Division of Psychiatry and Applied Psychology, School of Medicine, University of Nottingham, Nottingham, United Kingdom; ^15^Hassenfeld Children's Hospital at NYU Langone, New York University Child Study Center, New York, NY, United States; ^16^The ADHD Clinic, Manor Hospital, Oxford, United Kingdom; ^17^Genius Within, Plumpton Green, United Kingdom; ^18^Department of Organizational Psychology, Birkbeck College, University of London, London, United Kingdom; ^19^AADD-United Kingdom, Bristol, United Kingdom; ^20^Bristol Adult ADHD Support Group, Bristol, United Kingdom; ^21^Surrey and Borders Partnership NHS Foundation Trust, Leatherhead, United Kingdom; ^22^Department of Psychology, Institute of Psychiatry, Psychology and Neuroscience, King's College London, London, United Kingdom; ^23^ADHD Richmond and Kingston, London, United Kingdom; ^24^ADHD Solutions CIC, Leicester, United Kingdom; ^25^Cambridge & Peterborough NHS Foundation Trust, Cambridge, United Kingdom; ^26^ADHD and Psychiatry Services Limited, Liverpool, United Kingdom; ^27^Cheshire and Wirral Partnership NHS Foundation Trust, Chester, United Kingdom; ^28^University of Exeter Medical School, University of Exeter, Exeter, United Kingdom; ^29^ADHD Consultancy Limited, London, United Kingdom; ^30^Adult ADHD and Asperger's Team & Children and Young People's ADHD and ASD Service, Northamptonshire Healthcare NHS Foundation Trust, Kettering, United Kingdom; ^31^ADDmire Clinic, West Byfleet, United Kingdom; ^32^Epsom and St. Helier University Hospital, Epsom, United Kingdom; ^33^Cambridge Cognition, Cambridge, United Kingdom; ^34^Psychological Sciences, University of Bristol, Bristol, United Kingdom

**Keywords:** ADHD, service provision, healthcare commissioning, assessment, treatment

## Abstract

**Background:** Despite evidence-based national guidelines for ADHD in the United Kingdom (UK), ADHD is under-identified, under-diagnosed, and under-treated. Many seeking help for ADHD face prejudice, long waiting lists, and patchy or unavailable services, and are turning to service-user support groups and/or private healthcare for help.

**Methods:** A group of UK experts representing clinical and healthcare providers from public and private healthcare, academia, ADHD patient groups, educational, and occupational specialists, met to discuss shortfalls in ADHD service provision in the UK. Discussions explored causes of under-diagnosis, examined biases operating across referral, diagnosis and treatment, together with recommendations for resolving these matters.

**Results:** Cultural and structural barriers operate at all levels of the healthcare system, resulting in a de-prioritization of ADHD. Services for ADHD are insufficient in many regions, and problems with service provision have intensified as a result of the response to the COVID-19 pandemic. Research has established a range of adverse outcomes of untreated ADHD, and associated long-term personal, social, health and economic costs are high. The consensus group called for training of professionals who come into contact with people with ADHD, increased funding, commissioning and monitoring to improve service provision, and streamlined communication between health services to support better outcomes for people with ADHD.

**Conclusions:** Evidence-based national clinical guidelines for ADHD are not being met. People with ADHD should have access to healthcare free from discrimination, and in line with their legal rights. UK Governments and clinical and regulatory bodies must act urgently on this important public health issue.

## Background

Attention-deficit/hyperactivity disorder (ADHD) is a common neurodevelopmental disorder characterized by persistent and impairing inattention and/or hyperactivity-impulsivity (1, 2). ADHD usually first presents in childhood, and persists into adulthood in a sizeable proportion of cases (3, 4).

ADHD is common. Worldwide prevalence, estimated by standardized procedures in representative samples of the community, falls between 5 and 7% in children and adolescents (5, 6), and 2–4% in adults (7–9). Diagnostic and impairment criteria and source of information contribute to heterogeneity of estimates (6), and evidence suggests that using the same diagnostic criteria estimated prevalence rates vary significantly across different countries with rates being higher in high and high middle income countries (9). Research indicates significant genetic influences, environmental risk factors, and differences in pattern of brain correlates in affected individuals as shown in neuroimaging studies [reviewed in (10)].

ADHD is associated with a range of adverse outcomes. People with ADHD are more prone to accidents and injuries, and have a higher mortality rate compared to the rest of the population (11, 12). They are more likely to be involved in delinquency, criminal behavior and substance use (13, 14), experience early or unplanned pregnancy (15), and experience challenges in education and at work (16). Associated problems and comorbidities are common, frequently arise in childhood and are likely to accumulate during the lifetime; for example, research documents a trajectory of ADHD in childhood, leading to academic and social problems which, in some cases, leads on to depression (17).

Timely detection and treatment is likely to moderate risks and improve outcomes (18, 19). ADHD is commonly treated with psychostimulants, such as methylphenidate and amphetamine. A recent systematic review and network meta-analysis recommended methylphenidate for children and adolescents and amphetamines for adults, taking into account both efficacy and safety (20). Pharmaco-epidemiological studies show that during periods on treatment, people with ADHD have a lower risk of suicide (21), unintentional injury (11), motor vehicle accidents and substance use disorders (19), reduced hospital contact (22), better educational (23), and occupational (24) outcomes, as well as reduced criminality (22). Furthermore, evidence suggests delays in treatment lead to high long-term personal and public costs, including reduced economic productivity, and increased health, social care and state benefit costs (25, 26). Effective psychological interventions have been found to help increase employment and education rates and reduce use of cash benefits and social services (27).

There has been a stepped change in clinical policy for treating ADHD in the United Kingdom (UK) over the past three decades. This has co-occurred with the mainstreaming of childhood ADHD into generic mental health services, and the publication of the first national clinical guidance on adult ADHD by the National Institute for Clinical Excellence in 2008. As a result of these changes and increased recognition of ADHD in the population, there has been a significant increase in rates of first diagnosis and prescribing of childhood ADHD (28, 29), and many clinicians have seen their ADHD patient caseload and waiting list increase significantly.

However, ADHD and its treatment remains controversial in public, policy, and clinical spheres both in the UK (30–33) and in other countries. The controversy centers around the perception of ADHD as a medicalised social construct (34), represented in some newspapers as a catchall for naughty behavior [see for example (35)]. The increase in ADHD medication prescription in the UK over the last two decades has been a cause of national concern, provoking responses from health and educational representatives (36, 37). Concerns have also been expressed around the potential diversion and misuse of ADHD medication as a “study drug” (38). This leads to short-sighted calls to curtail prescriptions, but risks unfairly penalizing those who genuinely need ADHD medication.

However, contrary to concerns of over-medication, ADHD is more likely to be under-identified, under-diagnosed and under-treated in the UK (39, 40). Many of those who seek help face patchy, unavailable and inaccessible services, and extremely long waiting lists (41–43). Problems with access to services also affect young people with ADHD in transition from child to adult mental health services (44, 45). Patients report accumulated psychosocial burden from delays in diagnosis and treatment (46). Those who struggle to receive support are being signposted to, or are seeking out, local or national service-user support groups for help. These charitable organizations are inundated with support requests that they are not always equipped or qualified to fulfill, with certain regional exceptions. Those who are able to afford it turn away from the National Health Service (NHS) and toward private healthcare.

These problems have been exacerbated since the advent of the COVID-19 pandemic. Efforts to delay the spread of the virus have had an impact on demand and capacity to deliver support for people with mental health needs. The pandemic is associated with a range of social, financial, educational, health, and personal concerns, which are all stressors associated with mental health issues (47). Individuals with ADHD are likely to be particularly vulnerable to the distress caused by the pandemic and physical distancing measures, and may display increased behavioral responses (48). They may also be at greater risk of contracting COVID-19, a risk that appears to be exacerbated in ADHD patients who are untreated (49). Although these additional pressures on services have arisen more recently and are likely to increase with the exacerbation of clinical needs in this population, they have compounded already existing shortfalls.

Whilst acknowledging current challenges for mental health provision and the increase in service provision for ADHD over the past three decades, the existence of a significant unmet clinical need for individuals with ADHD in the UK demands scrutiny. With evidence of personal, clinical, social, and economic benefits of investing in adequate treatment for ADHD, access to clinical support must be improved not only in the context of the pandemic, but also beyond. It is on the basis of these key issues that professionals specializing in ADHD convened for a consensus meeting to discuss the gap in ADHD provision in the UK.

## Methods

The consensus group convened in London on the 11th February 2019. The meeting was hosted by three leading UK ADHD organizations; (1) the ADHD Foundation (https://www.adhdfoundation.org.uk); (2) The UK ADHD Partnership (UKAP, www.ukadhd.com) and; (3) the UK Adult ADHD Network (UKAAN, www.ukaan.org). Meeting attendees were academics, mental health professionals, educational and occupational specialists, service-user support services and charity workers specializing in ADHD. Healthcare practitioners represented both those working within private practice, and the National Health Service (NHS), the UK-wide universal healthcare system providing free or low-cost healthcare to UK residents.

The meeting commenced with presentations on (1) ADHD provision in the UK from the viewpoint of the ADHD Foundation, and (2) an overview of research on treatment and short- and long-term outcomes of ADHD. This was followed by a question and answer session, after which attendees separated into three breakout groups, in which discussions were facilitated by group leaders. Following the group work, all attendees re-assembled. Group leaders then presented findings to all meeting attendees for another round of discussion and debate, until consensus was reached.

The National Institute for Health and Care Excellence (NICE) guidelines provide clinical guidance for the diagnosis and treatment of ADHD within the NHS across England and Wales (50, 51), and adopted, as appropriate, in Northern Ireland (52). The Scottish Intercollegiate Guidelines Network (SIGN), provides the equivalent for Scotland (53). NICE guidelines were used as a benchmark for service provision in these discussions, since these provide official guidance for England and Wales and there is good overlap between NICE and other recommendations for the management of ADHD in SIGN.

Group discussions included the following three main topics, each of which was explored for differences between children and young people (age <18) and adults (age >18):

- How do we know children, young people and adults with ADHD are not being diagnosed and why is this happening?
- Are the NHS services provided adequate?
- What is happening to those with ADHD who cannot access NHS healthcare for their ADHD?

Presentations and debate amongst attendees were audio-recorded and transcribed. During group breakout meetings, a note-taker was allocated to each breakout group, and after the consensus meeting notes were circulated to participants in each breakout group for review. All materials (transcriptions, electronic slide presentations, and breakout group notes) were synthesized jointly by the lead author and writer.

Where relevant and available, consensus discussion points are provided alongside references to the supporting research literature, gray literature, policy or legislative documentation. Where reports are anecdotal only and relate to the clinical or professional experiences of consensus attendees, these are described as such in the following report. A final draft was circulated to all authors for approval before submission. The consensus outcomes therefore represent the views and recommendations of the authors in the consensus group as a whole, reflecting the views of a range of professionals involved in ADHD care and some key organizations working in this area.

## Results and Consensus Outcome

Shortfalls in detection and service provision can be inferred from discrepancies between community prevalence rates (defined as the rate of ADHD in a large sample of people in the general population) and administrative prevalence (defined as the proportion of people with a recorded clinical diagnosis) (39). Population prescription rates for ADHD medications are often used as a proxy for clinical diagnosis in administrative prevalence studies, but underestimate rates of diagnosis, since they do not take account of patients who are managed without ADHD medications.

When considering community prevalence studies (using similar methodological approaches) world estimates of the prevalence of childhood ADHD have not changed in the past three decades (54). By contrast, prescribing prevalence in the UK has increased over this period of time (28). This shows that rates of identification and access to treatment has improved over time, although these continue to remain low across all ages. Similarly, increases in rates of medication use for ADHD have been seen across a range of different countries worldwide over this period of time albeit with a broad discrepancy in treatment rates (40).

ADHD administrative prevalence (based on rates of diagnosis and/or prescriptions) in children and adolescents in the UK has been estimated to fall between 0.2 and 0.9% since the mid-2000s (39). These rates remain below community prevalence estimates in the UK estimated at around 2.2% in 1999 and 2005 (55, 56), with more recent estimates of 1.6% in 2017, based on the more restrictive ICD-10 Hyperkinetic Disorder criteria (57). Administrative prevalence of adult ADHD in the UK stands at around 0.1% (40), far below even some of the lowest prevalence rates documented in adults (9).

People with ADHD can face a long and difficult journey to reach diagnosis and long-term management, described in some cases as “an uphill struggle” (46). A number of hurdles to reaching treatment were highlighted during the consensus meeting and these are described in more detail in the sections below. Whilst pathways to care for children and adults are distinct, barriers to treatment are largely overlapping. After describing these barriers, we provide recommendations for improving provision and outcomes for people with ADHD.

### Barriers to ADHD Diagnosis and Treatment Provision

#### Detection of ADHD and Associated Problems

ADHD can only be diagnosed and treated as quickly as the condition is identified in the community. However, there are several reasons that it may go undetected. Expectations that ADHD expresses only as hyperactive, restless, and disruptive behavior may limit detection of the many more subtle presentations. Although more subtle inattentive problems can present across both sexes, they may disproportionately affect detection of ADHD in girls and women (58). Furthermore, lower rates of detection have been reported among minority racial/ethnic groups in the United States (59) and more research is required to examine for biases in operation for ADHD treatment in relation to ethnic and racial status in the UK.

ADHD symptoms in those with higher intellectual functioning and/or individuals applying a range of compensatory strategies to reduce or mask their difficulties may also go undetected. Even if detected, these individuals may not appear to meet impairment thresholds for referral to secondary health services and assessment.

Comorbidity is common and complicates identification and treatment in ADHD. Common comorbidities in children include autism spectrum disorders (ASD), mood, anxiety, oppositional, and conduct disorders, as well as specific learning and language disorders, epilepsy and Tourette's syndrome (60, 61). In adults with ADHD, comorbid symptoms and disorders are also extremely common (62) and include ASD, mood, anxiety, impulse control, and substance use disorders (63, 64). The consensus group noted that with increasing age, people with ADHD typically present with more co-occurring conditions, which can lead to diagnostic overshadowing, making diagnosis more complex and ADHD more likely to be missed. Symptomatic overlap with other disorders and a lack of awareness of ADHD in clinical practice can lead to diagnostic mis-specification (65, 66).

As children age and become more independent, their environmental support declines, whilst social, academic and environmental demands increase (67). There is now increasing evidence that in at least some cases, the full-blown disorder emerges during the middle adolescent years, when more demands are made on individuals and they fall further behind their peers (68). Some young people with ADHD may develop emotional dysregulation and comorbid disorders (for example, mood disorders, eating disorders, and self-harm), triggering inclusion into different treatment pathways.

People with ADHD may come in contact with a range of mental health, social care or criminal justice professionals in their lifetime. However, ADHD often remains unrecognized and provisions are put in place for other conditions. This is evidenced by the high rates of unrecognized ADHD in patient populations treated for other psychiatric conditions [15.8–17.4% (69)], and in prison populations [25.5% (70)]. Importantly, the consensus group noted that adults with ADHD may show poor response to the treatment of comorbid conditions if ADHD symptoms are not appropriately managed. Symptoms such as emotional instability, characteristic of many other mental health disorders, often improve when ADHD is treated (71).

ADHD symptoms tend to decline with increasing age (7), with greater decline seen for hyperactive-impulsive symptoms, but less so for inattentive symptoms (72). Adults with ADHD can therefore have a more subtle presentation characterized by more internalized symptoms rather than overt externalized behavior (1). The consensus group noted that with increasing age people with ADHD may present with a variety of additional difficulties (e.g., insomnia, anxiety, depression), and are less likely to attribute problems to ADHD. In older adulthood there may be confusion between the lifelong attentional problems of people with undiagnosed ADHD and prodromal dementia (73). The experience of adult mental health clinicians in the group was that most patients presenting in adult ADHD clinics for the first time had not previously received an assessment or diagnosis for ADHD as children.

#### Gatekeepers of ADHD Assessment and Diagnosis

In the UK healthcare system, a patient with ADHD passes through multiple stages in the process of seeking help. The knowledge and attitudes of the network of gatekeepers can facilitate or hinder their access to support. One problem noted repeatedly during the consensus meeting is that there is no consistent referral route across all regions of the UK.

Parents/carers are often the first to seek out referral and diagnosis for their children and their perception of the presenting problem is a key contributor to primary care referrals to community pediatric and/or Child and Adolescent Mental Health Services (CAMHS) (33, 74). Similarly, the understanding and attitude toward ADHD of help-seeking adults is likely to affect their likelihood of self-referral.

Teachers are often the first (and may be the only) professionals approached by parents or carers (74). Teachers and educators are also in regular contact with a large number of children and young people and they are well-positioned to identify when a child or young person is struggling. However, teachers are more likely to raise concerns regarding ADHD when the child presents with notable hyperactivity-impulsivity and associated disruptive behavior (75). Furthermore, referral may be hindered by overemphasizing the role of adverse home environments and dietary factors as primary causes of symptoms (76) and/or the perception that poor parenting is to blame (77). With the diagnostic requirement that ADHD symptoms are pervasive and present across two or more settings, school observations and teacher input can support or undermine a legitimate referral for assessment and diagnosis.

Primary care services are another main point of contact for parents/carers and are usually the first port of call for adults for onward referral for ADHD assessments. Unfortunately, some primary and even some secondary care physicians express uncertainty about the legitimacy of an ADHD diagnosis (78, 79). They may have negative and unhelpful attitudes about ADHD (46), and may perceive parental help-seeking as reflecting the desire to “shift blame” or find a “quick fix” for behavioral or disciplinary problems (80). As a result, some affected adults, young people and their families may experience blame or dismissal (30, 79).

Progressive revisions to diagnostic criteria and clinical practice over the last 30 years have broadened the ADHD phenotype. Some healthcare practitioners may not be aware of these changes or may have limited “buy-in” (81), and still be discounting a variety of ADHD presentations, such as adult ADHD, ADHD comorbid with ASD, or inattentive only presentations. These presentations may be met with an extra dose of distrust, particularly for adult ADHD for which the consensus group noted stigma remains particularly high.

#### ADHD Healthcare Organization in the UK

The consensus group noted that there are local and regional idiosyncrasies in referral pathways and treatment arrangements for both child and adult ADHD services, which can make it challenging to navigate access to care.

Insight into health service provision in the UK can be obtained from public authorities through legislative rights under the Freedom of Information (FOI) act of 2000 (82), which allows access to information on the daily workings of public services. However, the information provided may not be complete and a non-response may reflect a reluctance to report on gaps in services or other constraints, such as lack of time or staff to respond (83). Statistics such as waiting times can be misleading due to, for example, closure of referrals and waiting lists once services reach capacity (84), or due to internal triaging where there is an initial waiting list for a generic assessment followed by yet another waiting list for ADHD assessment in patients with suspected ADHD. This practice of undisclosed data manipulation, can make the FOI data provided impossible to interpret straightforwardly and more difficult still to compare across services or regions.

##### ADHD Service Pathways in Children

At primary school age, ADHD is diagnosed and treated by Developmental Pediatricians in some regions or CAMHS in others; at secondary school-age it is usually managed through generic mental health services for children and young people [Community Child Health (CCH) or CAMHS]; in older teenagers and in adulthood the service is provided by specialist ADHD services or Adult Mental Health Services (AMHS) with expertise in ADHD.

After diagnosis and medication titration (where indicated) in secondary care, clinical care is often, but not always, transferred back to primary health through shared care protocols. Where this is the case, GPs assume responsibility for prescribing and providing routine physical check-ups. NICE then recommends that annual reviews of ADHD medication be conducted by healthcare professionals with training and expertise in managing ADHD (50).

A report from the Education Policy Institute using data from FOI requests from CAMHS Services revealed a “postcode lottery” for access to general mental healthcare. Median waiting times ranged from 1 day to 6 months and there was wide variation in the rates of referral rejection, with specialist mental health services rejecting referrals from as many as one in four children (24.2%) in 2017/18 (41). The most common justification for rejection was that the referred mental health conditions were not serious enough to meet the eligibility criteria for treatment, which included young people who had self-harmed or experienced abuse. Whilst these statistics do not reflect wait times and rejection rates for ADHD in particular, they highlight problems with mental health provision across CAMHS. Long waiting times were also reported in a 2016 survey by the Royal College of Pediatrics and Child Health. They reported that the average time from referral to diagnosis for ADHD exceeded 6 months for Community Child Health teams (42, 43).

The consensus group discussed the erratic way in which exclusionary criteria from service provision is applied. From the experience of the consensus group, some services only accept children and young people with ADHD when a patient presents with comorbidity. Others only accept those presenting with acute comorbidity (such as self-harming behaviors or eating disorders). Charity representatives spoke of instances when access to services had been declined to children as young as 14 years due to waiting lists that were so long the child would exceed the age cut-off for the service before they were seen. Anecdotal accounts were provided of young people with ADHD and severe comorbidities (self-harm, eating disorders, attempted suicide) being refused treatment for these comorbidities, on the basis that they were attributed to impulsivity (rather than a reflection of mental distress and an intention to self-harm) and their existing diagnosis of ADHD. The effect of these policies is that vulnerable young people and families seeking support are turned away, refused access to healthcare, and do not receive any support at all.

##### ADHD Service Pathways in Adults

**Figure 1:**
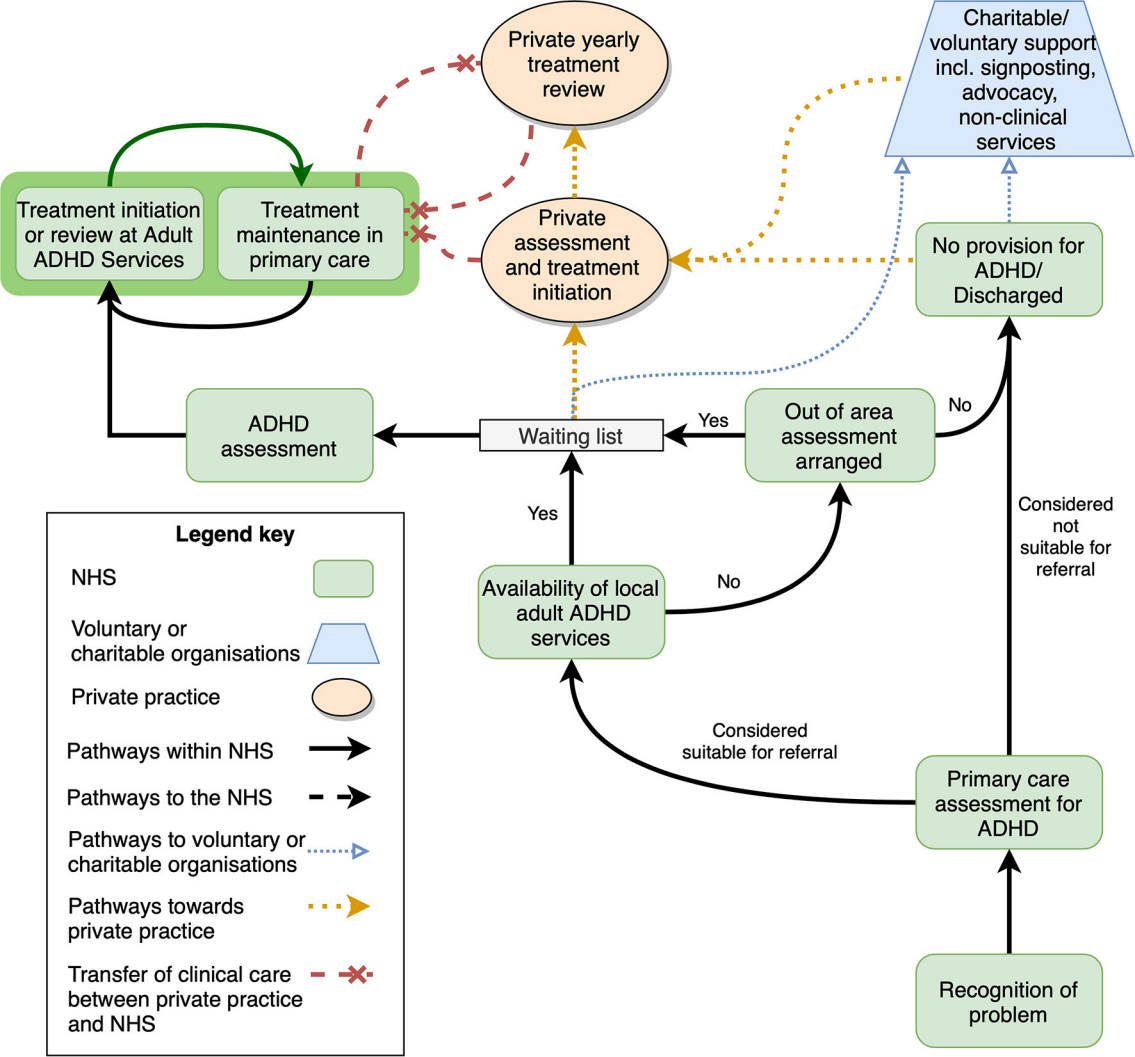
Successful and unsuccessful pathways to Adult ADHD treatment initiation and maintenance. This simplified schema shows the interaction between NHS bodies and services, voluntary and charitable organizations, and private health services. Shared care arrangements are shown on a dark green background. Note that in the experience of the consensus group, shared care between NHS and private practices (red dashed line) are infrequently supported. NHS, national health services; ADHD, attention-deficit/hyperactivity disorder.

Evidence suggests a similar “postcode lottery” for access to adult ADHD services, albeit with even longer waiting lists. A national survey found patchy provision of services for adults with ADHD (85), and in some localities services simply did not exist at all. In regions without adult ADHD specialist services, individuals should, by right under the NHS Constitution, be able to access these services elsewhere (86), however service commissioners may delay or refuse to fund out-of-area ADHD treatment (87). As a result, some people with ADHD are left in limbo, unable to access clinical care or social support, and unable to benefit from their legal rights and support systems associated with their disability. Help-seeking pathways for those suspecting a diagnosis of adult ADHD are shown in Figure 1. These pathways do not reflect how the healthcare systems should be structured, but rather provide an overview of the complex pathways that patients may take when seeking support for ADHD. Furthermore, this simplified schema shows key points at which patients may seek out help external to the NHS, either from voluntary or charitable organizations or from private health services.

Clinical Commissioning Groups (CCGs) are regional bodies of the NHS, which allocate, plan and provide services for populations within specific service regions. A review of ADHD provision in CCGs in England was undertaken in 2018 with Freedom of Information (FOI) requests by Takeda pharmaceuticals and the report was supported and endorsed by the ADHD Foundation charity (88). This report revealed a lack of oversight for demographic need for ADHD services by CCGs: only around one-third of CCG respondents provided information about waiting times or budget spent on ADHD services; less than one-third gave an estimate of the number of ADHD patients for whom they commissioned services. Where information was provided regarding the average waiting time from referral to assessment, this spanned from 4 weeks to 3.8 years (average 14 months). In July 2020, BBC News reported FOI data on adult ADHD waiting lists from 33 NHS trusts. A total of 20,859 adults were on waiting lists for ADHD services; 11 trusts had maximal waiting times of at least 2 years; and some reported waiting times of over five (89).

Internal reports from NHS governing bodies in certain parts of the country show the tensions that arise between the desire to reduce long patient waiting times, the likely additional costs from increased service investment and resultant increased prescription rates (90, 91). These conflicts may effectively paralyze progress in terms of increasing service delivery for affected patients. Financial constraints within individual services reveal the myopic nature of treatment and commissioning arrangements. This is particularly true for people with ADHD where treatment shortfalls are associated with significantly increased societal and personal costs, and which are shouldered elsewhere in the health, social care and judicial systems.

#### Barriers to Treatment

NICE guidelines emphasize the importance of recognition of ADHD, diagnosis and treatment, continuity of care, and ensuring that people with ADHD have a comprehensive, holistic shared treatment plan that addresses psychological, behavioral, and occupational or educational needs (50). However, access to treatment is not always straightforward, even for those who are already diagnosed. The consensus group identified four key areas of concern, outlined below.

##### Lost in Transition

Transition in healthcare refers to the process of transferring the clinical care of a patient from child to adult services, which occurs by the age of 18 years in most cases. There are multiple possible transition pathways in the NHS which vary regionally, and include referral to Adult Mental Health Services (AMHS), to specialist ADHD services or to GPs in primary care.

NICE guidelines recommend that young people with ADHD receiving treatment from CAMHS or pediatric services should be assessed at transition age, and then if needed transferred to adult services, where they should be re-assessed (50). Transfer of care should occur alongside a handover of full information, formal meetings between child and adult services, with planning of transition including the young person (50). Specific guidance is available regarding the implementation of NICE guidelines to achieve successful transition between services for young people with ADHD (92). However, in practice NICE guidelines on transition are often not implemented due to resource and time constraints and the multiple structural and organizational barriers in which service providers operate (78, 93, 94).

Transition takes place at a critical juncture in young people's lives and there are understandable concerns about the effects of sudden treatment cessation on their educational and/or occupational outcomes (95). One study of prescribing rates showed that rates of primary care prescribing of ADHD medication for young people in the UK declined more steeply than expected given the rate of symptom reduction—suggesting that some may experience cessation of medication due to a change of services rather than due to symptoms declining (96).

The consensus group noted that treatment cessation may arise from adolescents electively disengaging with clinical services. Others may be discharged or lost to follow-up during the transition stage for a range of reasons: a lack of adult services to which they can be referred, not meeting criteria for inclusion in adult service criteria (severity or impairment); a lack of knowledge or resources for treating ADHD within available services; and failure to attend the first post transition appointment (78, 93, 97–99). The CATCh-uS study prospectively examined transition into adult services in young people with ADHD in the UK, and found that only 1 in 5 eligible young people transitioned successfully (99).

When transition to adult ADHD services is unsuccessful, child mental health services may be left with the responsibility of continuing care, further decreasing their capacity to respond to new referrals (100). In some regions, ADHD services for children are supported entirely by CAMHS without involvement community pediatric services, and with limited availability of shared care prescribing with primary care services. On transition, primary care providers are suddenly expected to take over the unfamiliar role of long-term routine monitoring of ADHD medication in these same patients, without specialist oversight as recommended in NICE guidelines, and may refuse. As a result, young people with ADHD may suddenly and unexpectedly lose access to their usual treatments.

In cases where treatment is stopped during adolescent years, the consensus group noted that it can be difficult for young people to restart treatment again.

##### Lost in Communication

NICE guidelines state that ADHD diagnosis and medication initiation and titration should be conducted by a specialist in ADHD (50). Once patients have been stabilized on medication, treatment can be managed and monitored jointly with primary care, under a shared care protocol. Specialist review is recommended on an annual basis, but routine check-ups and prescribing can be completed in primary health. This frees up some capacity within secondary health services, allowing them to take on new referrals and manage more complicated cases.

However, often shared care arrangements fall apart, seriously disadvantaging patients caught in the middle. This can be due to lack of agreement or inadequate communication, or where primary care practitioners are concerned about assuming responsibility for an unfamiliar treatment (101). Insufficient support from secondary healthcare services may undermine continuity of care for affected patients. For example, a Royal College of Pediatrics and Child Health report published in 2017 showed that only 11.4% of Community Child Health (CCH) services could always see ADHD patients when follow-up was due, and 60% could do so no more than half the time, raising issues of medication safety (43).

Primary care practitioners may feel that ADHD symptom monitoring is not in their remit, and some believe that prescribing for ADHD should not be carried out by primary care (80, 102). Reasons for not prescribing include concerns regarding the diagnosis, the unavailability of non-pharmacological treatment, and potential inadequacy of physical monitoring in secondary care (102). Primary care practitioners also voice concerns around the nature of medication in terms of stimulants being “controlled drugs,” and risk of potential misuse and diversion (103), they may lack confidence in managing ADHD treatment, due to knowledge limitations, insufficient training and/or poor communication from specialist services (104). In a survey of 150 General Practitioners (GPs), 64% stated that they would be likely to change their views on prescribing with clearer advice from specialists, and 67% said they would be influenced if there was a clear protocol for monitoring a child on medication (104).

Problems with shared care may lead to patients navigating a complex system of referral for assessment, obtaining a diagnosis and initiating treatments under a specialist (sometimes after many years of seeking treatment), and then having treatment denied due to the failure of shared care.

As well as posing a risk to the safety, health and well-being of patients, breakdown of treatment provision due to failure of shared care represents a waste of health resources that are already in short supply.

##### Non-pharmacological Treatment

Pharmacotherapy for ADHD as a standalone treatment is unlikely to address the needs and impairments associated with ADHD fully (105). It does not provide direct support for common problems associated with ADHD, such as psychosocial, emotional and peer problems, behavioral difficulties and other comorbid conditions. A range of non-pharmacological approaches may be helpful working in cooperation with patients themselves, schools, parents/carers, educational and/or workplace settings. These include (provided in individual and/or group format) cognitive behavioral therapy (CBT), psychoeducation, parent mediated/training interventions, school/classroom interventions and occupational therapy (105). Although some commissioners and healthcare practitioners recognize the value of multimodal treatments consisting of non-pharmacological interventions alongside medication (106), psychological therapies for ADHD are often not implemented because they are considered to be expensive, because they are in short supply or simply not available.

For children and young people, treatment options are often rigid in nature, and not tailored to patients' needs. Parent/carer training interventions are the main (and sometimes only) option. However, since many of these interventions have been developed for children with externalizing and conduct problems (107), they are not always appropriate for supporting the needs teenagers and young people with ADHD (105) who require more direct support, and those with inattentive symptoms or subtler or more nuanced presentations.

Psychological interventions are rarely available for adults with ADHD, and medication is often the only treatment offered. A recent mapping study found that only 12 out of 44 dedicated adult ADHD services (27%) provided the full range of treatments recommended by NICE including psychological therapy (85). The consensus group noted that adults with ADHD may be more likely to be offered non-pharmacological treatments for coexisting conditions, although these frequently do not take ADHD and its associated difficulties into account.

#### Plugging the Gaps

When faced with long waiting times, patchy and unavailable services, blame or dismissal, affected individuals and their families may turn to other services and sectors to gain the support they need (see Figure 1).

Charitable and other support organizations provide a range of invaluable non-clinical services for people with ADHD at low cost or free of charge. These services include: information dissemination, signposting, peer and social support, friendship and preventing loneliness, advice clinics, coaching and psychoeducation, group and family therapy, and parenting programmes. Their websites provide access to freely available curated resources and/or help to direct people to relevant tools and support information. Services are not regulated however, and availability, resources and expertise vary considerably between areas and organizations, giving uneven coverage across the UK.

Support group representatives at the consensus meeting reported that patients are seeking out (and in some cases being actively directed to) local or national support groups for help. Informal referrals from the NHS led them to feel like a “secondary NHS” service, inundated with support requests that they felt ill-equipped and unqualified to fulfill. Their staff do not always have the training or expertise to support the vulnerable, distressed and at times suicidal individuals with ADHD, for whom help from clinical health services is delayed or refused.

Some affected individuals and families are driven to seek out costly private clinical services (26). For many, this is not an option for financial reasons. This leads to a 2-tier health service, unfairly penalizing lower income families and patients and leading to a gap in provision. Clinical assessment and treatment in the private sector can be costly. This is not a “one off” assessment service as it often requires further titration appointments, (repeat) prescriptions and non-pharmacological services. These can become unaffordable in the longer-term even for middle income patients and families, particularly for those with more complex clinical presentation and treatment needs.

Some patients and families seek only ADHD assessment in private clinics in order to circumvent gaps or blockages in access to care, in the hope of reintegrating into public healthcare provision by returning to the NHS (e.g., Figure 1). However, there is an absence of transparency regarding the expertise in ADHD and the quality of care provided in the private sector. NHS providers may have reasonable concerns regarding the validity of certain privately formulated diagnoses, which may leave NHS providers with no choice but to refuse treatment, leaving affected patients unable to access the support that they need.

Concerns around the quality of assessments and clinical reports for ADHD were an area of serious debate during the consensus meeting. Consensus participants (including private and NHS clinicians, stakeholders and support group representatives) reported problems with private healthcare providers capitalizing on the desperation of patients who feel let down by the public healthcare system. The upshot is that some patients seeking out private services for ADHD do not receive appropriate assessments or treatment that follow clinical guidelines. In some cases, private ADHD assessment reports do not contain enough information to show that diagnostic thresholds had been met or do not clarify if the required in-depth examination was carried out, and therefore patients' diagnoses cannot be accepted at other clinics until they are given a more comprehensive assessment. Consensus participants reported cases of ADHD diagnostic reports written by assessors without appropriate clinical qualifications (i.e., those without core clinical training in assessing differential diagnosis and/or comorbid conditions) and/or who are not registered with a UK health regulator (e.g., Health and Care Professionals Council, General Medical Council).

### Recommendations

The consensus group identified essential changes that can and should be made to improve outcomes for people affected by ADHD. Solutions include training and education across all sectors; revising models of care to improve access to treatment; a focus on joining up services; methods to improve consistency in diagnosis and treatment across service sectors; broadening access to non-pharmacological treatments; and funding and commissioning for ADHD.

#### Training and Education Across All Sectors

Access to evidence-based training will address the issue of awareness and attitudes of key professional groups in the public sector and improve recognition and support for individuals with ADHD. Ideally, this should reach all healthcare professionals (including primary care providers), educational professionals, youth center employees, social workers, the police, and those working with prisoners or youth offenders.

Training should incorporate heterogeneity of ADHD (described in section Detection of ADHD and associated problems), to improve detection of more subtle presentations. Key professionals need to be informed of the biological and medical evidence to help shift blame away from parenting and the home environment. An emphasis on longer-term outcomes of effective treatment may help to reduce the perception that those seeking ADHD treatment are looking for a “quick fix.”

ADHD awareness for educational professionals should be developed for schools and higher education institutions to improve referrals from the education sector. Where possible, educational psychologists or mental health professionals working in schools should be engaged to assist with development and implementation of training.

#### Empowering Primary and Secondary Care in the Management of Adult ADHD

The consensus group agreed that competency in ADHD should ideally occur alongside competency for other common mental health conditions: for clinicians already diagnosing and treating anxiety and depression, concurrent assessment and treatment for ADHD (where appropriate) would improve the targeting of treatments, lead to better outcomes, and reduce patient and clinical burden. There should be adequate knowledge and specialism of ADHD within all secondary mental health practices, as there is for other common mental health conditions.

In accordance with NICE guidelines (108), a broad range of health professionals (psychiatrist, pediatrician, clinical psychologist or other appropriately qualified healthcare professional) can with adequate training, acquire the necessary knowledge of ADHD to support its assessment and/or treatment.

Primary care physicians, including general practitioners (GPs), already have an important role in raising suspicion for a possible diagnosis, making a timely referral and supporting monitoring and continuation of treatment under shared care protocols. Providing, and encouraging primary care practitioners to participate in, training about ADHD was considered to be an important first step, and encouraging some GPs and practice nurses in taking on additional training to qualify as GPs with extended roles (GPwERs) or specialist nurses with extended roles, respectively, was considered practical and sustainable for improving expertise in in primary care in the first instance. Overall, the group was in agreement that a level of competence in the recognition and understanding of ADHD was key, as well as clear pathways to more specialist support.

In the longer term, integration of ADHD into broader mental healthcare provision in primary care would be required to further streamline provision and remove bottlenecks. However, this arrangement has implications for GP workloads, and would require additional workforce management, staff and resource provision. The new Primary Care Network model, described in the NHS Long Term Plan in 2019 (109), aims to pool expertise at a primary care level within local areas. Primary care at scale, with practices joining together to pool expertise and resources in larger primary care networks with 30,000–50,000 patients, may help to address implications for workforce management and resource provision.

#### Joining Up Services

We have shown a disjoint between primary and secondary care, and child and adult services, which leaves patients with ADHD unable to access or maintain treatment. Improved communication is key to joining up services. When primary care services are asked to take over prescribing from secondary health providers there needs to be support and guidance available for treating clinicians, with specialist advice readily available. Examples of integrated care between GPs and specialist services for chronic illnesses such as heart failure have been reported previously, resulting in improved coordination, GPs feeling more confident in supporting their patients, specialists receiving more detailed feedback from primary care, and patients themselves benefiting from more streamlined and holistic care (110).

Longer-term, staff with specialist or extended roles within primary care networks, as described above, could help to close the gap in accessing diagnostic and intervention services, and liaise with and between secondary care services and psychological services as needed to provide the correct intensity of support and the required continuity of care.

#### Improving Consistency in Diagnosis and Treatment

Consensus meeting discussions highlighted the importance of regulating private ADHD practices to improve consistency of ADHD diagnosis, assessment reporting and treatment, and to ensure adequate qualification of providers.

The consensus group also noted varying quality in ADHD assessments within the NHS, albeit to a lesser extent. These problems led to discussions within the consensus group on the minimum standards for an ADHD diagnostic assessment. These were agreed on and are summarized in Table 1. Adhering to minimum diagnostic standards may help to support an ADHD diagnosis obtained in private healthcare for those wishing to return to NHS treatment. These standards can also help patients to understand which investigations to expect from an ADHD assessment, and what to expect from their diagnostic report, and provide confidence in their diagnosis. Patients/clinicians can also examine reports from Care Quality Commission in the UK, which monitors, inspects and regulates healthcare services, including private and NHS healthcare services. Inspection reports can provide an initial overview of the quality of individual private services, and are publicly available on the Care Quality Commission website.

**Table 1:** Minimum standards for ADHD assessment and report.

ADHD diagnostic assessments should be of an adequate length to cover all aspects described below and provide detail and generate examples of behaviors or problems. The clinician completing the assessment should be highly familiar with and/or specialize in ADHD but will require specialism beyond ADHD to identify comorbidities and complexities. Patients should not usually expect to receive their diagnostic report on the same day as their assessment.
**Assessments should include:**
• *Structured clinical interview* according to up-to-date DSM or ICD publications, including:
° Developmental history
° Medical and physical health history
° General psychiatric history
° Family history
° Educational/occupational history
° Impairments
° Exploration of potential comorbid problems/differential diagnosis
° Risk assessment
• *Collateral information to inform assessment, if available, can be used to augment clinical decision making and should not be used as stand-alone diagnostic tools*. e.g., rating scales, information from informants (including schools), school and/or other reports, and objective tests of attention, impulsivity and/or motor activity.
• *Mental state examination*
• *Physical assessment*: physical observation, pulse, blood pressure, height and weight, cardiovascular assessment, and referral for other types of physical examination if indicated.
**Diagnostic reports should include:**
• Description of diagnostic assessment completed (e.g., measures, corroborating information)
• Clear diagnosis and formulation.
• Outline of symptoms and impairments
• Coexisting diagnosis and associated problems
Risks
• An outline of strengths in the assessment report
• Individualized recommendations, including treatment plans (pharmacological, non-pharmacological, multi-agency liaison).
• Contact details for local service-user support services

#### Improving Access to Non-pharmacological Treatment

The consensus group agreed that access to non-pharmacological treatment should be improved for patients with ADHD. Treatment options should include a broader range of non-pharmacological interventions, tailored to developmental age and need.

In accord with NICE guidelines, the group emphasized the importance of access to high-quality age-appropriate psychoeducation for everyone with a new diagnosis of ADHD and their families. Evidence suggests that psychoeducation may help to improve clinical and subjective outcomes as well as medication adherence (111). Some initial psychoeducation should be provided at the point of diagnosis of ADHD by the diagnosing clinician. Further enhanced psychoeducation should be tiered based on severity and complexity of patient needs and consider comorbidity.

In children and young people, age- and presentation-appropriate psychological interventions (beyond parent/carer training for behavioral problems) should be made available in line with NICE guidelines.

Psychological interventions should also be increased for adults with ADHD. Aside from symptom management, these should address the specific difficulties experienced by young people and adults with ADHD (e.g., emotional lability and dysregulation, educational and employment problems, interpersonal difficulties, antisocial behavior and the development of prosocial competence, substance misuse, self-harm, dysfunctional coping strategies, and comorbid conditions such as anxiety and depression). Services such as IAPT (Improving Access to Psychological Therapies), which provide psychological therapies for adults with anxiety and depression in the community, could be useful resources for adults with ADHD if opened up or adapted for this patient group.

#### Funding and Commissioning for ADHD

ADHD is a common mental health condition. However, this contrasts to the clinical provision model, where diagnosis, treatment initiation and monitoring is constrained to scarce and limited capacity specialist health resources. The experience of the consensus group was that services struggle with the capacity vs. demand conundrum, and that many service providers are passionate about providing the best support that circumstances and local commissioning parameters allow.

The rights of people with ADHD are supported by the UK Equality Act (112). Clear guidance on clinical practice to support healthcare in ADHD is spelled out in national clinical guidelines. NICE guidance is linked to each CCG's responsibility and legal duty to regard NICE quality standards and recommendations, secure high quality services and ensure continual improvement in the quality of local NHS services (in addition to their legal duty to reduce health inequalities) as set out in the NHS Constitution (113) and The National Health Service Act (2006) (114) as amended by The Health and Social Care Act (2012) (115). Furthermore, it appears that courts are increasingly willing to acknowledge that national guidance may be relevant (in conjunction with clinical judgement) in setting standards of care because they are evidence based and reflect reasonable medical practice (116). This means CCGs and clinicians are potentially at risk of being challenged if they ignore NICE Guidance and they should only do that if they have something better to offer.

The rights of people with ADHD in the UK are also protected under the Human Rights Act 1998 (article 14: right to non-discrimination), and further under the UK Equality Act 2010, which protects people with a disability (including ADHD). People with ADHD also have rights under the Public Sector Equality Duty in England, Scotland and Wales, which places an obligation on public authorities to positively promote equality, not merely to avoid discrimination.

However, the existence of these clinical guidelines and the legislation to underpin them has not hindered people in key positions for referral, diagnosis and service commissioning from denying access to treatment and support to affected individuals. It is also clear that some CCGs are not commissioning or staffing adequate services for ADHD, and are therefore disregarding clinical evidence, national clinical guidelines, and their legal duty to prevent health inequality and discrimination. This problem is most clearly shown for adults with ADHD, for whom services are simply not available in certain regions, or to whom access to NHS services in other areas are not made available. In order for UK-wide clinical guidelines to be meaningful and effectively implemented, funding and service development needs to increase to meet the burden of illness.

The fragmentation of funding across public services in the UK means that it can be difficult to convince health commissioners to see ADHD treatment as a broader investment in health, education, social services, the criminal justice system and the economy in general. Longer-term benefits are also a hard sell for commissioners who work toward a fixed annual budget, with competing needs from different mental health groups and services that are already over-stretched. Commissioners must be informed on the robust evidence for the long-term negative outcomes associated with ADHD (especially if untreated) and their economic implications (25).

Long-term planning of funding (now implemented in some regions in the UK) is needed to circumvent problems arising from “short-termism” of care. Joined-up commissioning between state-funded health, social services, and judicial services may help to reduce fragmentation of care and cost burdens.

## Discussion

ADHD is the most highly prevalent childhood condition and is also common in adults. There is robust evidence for negative health and social outcomes. Yet many young people and adults are not able to obtain a timely diagnosis and treatment. Clinical guidelines are not being implemented and there is huge variation in the commissioning of ADHD services across the UK.

The current paper highlights discrepancies between clinical guidelines and provision in key areas (e.g., basic service availability and access, transition from child to adult services, shared care, non-pharmacological treatment). These discrepancies occur at multiple points in the healthcare system and often have complex socio-cultural, health-structural, funding, commissioning, and resource-based underpinnings. However, despite the underlying complexity of the system, it must be said that unmet needs correspond to denied rights, particularly in a universal health service.

The greatest barrier to services for people with ADHD is lack of awareness and stigma associated with the ADHD diagnosis. From the patient perspective, stigma and lack of recognition of the condition create a barrier to accessing treatment and support systems. From a commissioning and service delivery standpoint, lack of understanding and stigma constrict allocation of funding and resources. As the current report shows, prejudice and lack of understanding influence all levels of the healthcare system, resulting in a de-prioritization of ADHD and its treatment. This also has an effect on the physical and mental health and well-being of patients and their families who struggle to access the support they are entitled to and face ill-placed blame. Delays and inability to obtain treatment lead to increasing morbidity and functional impairment.

Most importantly, we need to end acceptance of stigma against people with ADHD as a valid viewpoint in the healthcare sector. The growing strength of vocal and active support groups and charities across the country have provided an emergent patient voice and public visibility of the condition has improved. Widely publicized reports produced by, or in association with, these charities and support groups have also helped to raise the profile of ADHD and associated problems (26, 88), and positively influence the nature of reporting around the condition.

We should now be looking toward improving access to treatment for children and adults with ADHD. A brief overview and summary of the consensus recommendations is presented in Table 2.

**Table 2:** Overview and recommendations.

• There is an urgent need to tackle the underlying structural, social, and economic restrictions that de-prioritize mental health and ADHD in healthcare.
• Training for ADHD should be provided across disciplines and sectors to reduce stigma and misinformation and improve detection.
• Longer-term planning and budgeting are required to provide a “whole person” approach and reduce short-termism and fragmentation of care.
• Devolving of health and social care within one budget can help to reduce service fragmentation.
• Additional efforts and investment are needed to join up components of clinical service delivery from child to adult services, and between secondary and primary health.
• Current healthcare provision for ADHD in the UK is overly complex and regionally variable. We need to look toward new models of integrated care to provide more streamlined and effective neurodevelopmental services.
• ADHD should be viewed as part of common adult mental health, rather than a specialist diagnosis. Due to its high prevalence, and high comorbidity with other mental health conditions, adult ADHD should be mainstreamed into secondary care.
• Reinforcement for ADHD services from primary care is likely to be needed to support treatment in the longer-term.

Information provision and training for key professional groups, including healthcare and educational professionals, could lead to more timely and appropriate referrals, assessment and treatment. Reducing stigma and increasing understanding of ADHD may help to improve understanding and increase referrals to clinical services.

Whilst the rights of individuals with ADHD are strong under current UK legislation and existing clinical guidelines, accountability of services and service commissioners to these rights and guidelines is uneven. Greater regulatory and legislative support for ADHD could go a long way toward reducing stigma and opening up pathways to healthcare. In 2018 an All-Party Parliamentary Group for ADHD was launched at the Houses of Parliament. Whilst this provided a positive platform there have yet to be any tangible outcomes. In 2017 the charity ADHD Action called upon the Government to pass an ADHD Act that would meet the needs of adults and children with ADHD (117). Their petition received 11,806 signatures but was not supported by the government. A second petition was recently launched (118), currently reaching over 10,000 signatures.

However, legislation and regulation alone will not ease the problems of overly complex, overstretched and fragmented services. Mainstreaming ADHD provision into general adult mental health services, and introducing new expertise within primary care across child and adult ADHD healthcare provision can help to improve detection, take the pressure away from over-stretched specialist services, and enhance communication throughout the healthcare system. Enhancing information flow between primary and secondary care services, and child and adult services can help to reduce the likelihood that diagnosed patients find themselves falling between the edges of service boundaries, unable to access the treatment they need.

Whilst implementing the above recommendations will entail additional costs to the UK healthcare system in the short term, economic analyses indicate that leaving ADHD untreated or undetected is not a cost-saving exercise. Evidence suggests delays in effective treatment lead to high long-term personal and public costs, including reduced economic productivity, and increased health, social care and state benefit costs (25, 26). Further investment in mental health in general, and ADHD in particular, is required to support the services which are groaning under the weight of demand.

As we move toward resuming mental health services in the post COVID-19 era, we must look to how we can improve access and treatment in the future. Guidelines now exist on ADHD management and treatment initiation during the COVID-19 pandemic (48, 119). Much can be achieved at distance using digital communications, which can help clinics to resume patient contact, assessment and treatment monitoring. However, these are merely short-term solutions that alleviate problems at the tip of the iceberg. Much larger changes, in terms of workforce education, service delivery modeling and financial investment are needed to resolve the broader issues described.

## Data Availability Statement

The original contributions presented in the study are included in the article/supplementary material, further inquiries can be directed to the corresponding author.

## Author Contributions

SY, PA, and TL were responsible for the planning and scientific input of this consensus statement. All authors (except CS and AL) attended the consensus meeting. CS completed the first draft of the manuscript. This was reviewed by SY, who also consulted further with some authors about specific points. A second draft was then generated jointly by SY and CS and circulated to all authors for comment. Following further amendments, a final draft was circulated for comment and endorsement of the consensus. All authors have read and approved the final manuscript.

## Conflict of Interest

SY, SCu, BZ, CJ, HR, ND, AL, WC, and PM have pecuniary affiliations with consultancy organizations and/or private practices. CS was employed by the company Cambridge Cognition. SY received honoraria for consultancy and educational talks years from Janssen, HB Pharma and/or Shire. She is author of the ADHD Child Evaluation (ACE) and ACE+ for adults. She is a consultant at the Cognitive Center of Canada, who publish R&R2 for ADHD Youths and Adults. PM received honoraria for consultancy and educational talks from Shire, Takeda, and Flynn Pharma. KvR received honoraria for educational talks from Shire/Takeda, Lilly, Janssen, Medice, and Flynn. CJ provided educational talks for Lilly and Janssen. MP received honoraria for talks and advisory board participation, and travel support for conference attendance, from Shire/Takeda & Flynn Pharma. TN-D reported travel and consultation fees paid for attendance at Transition into Adulthood ADHD (TiAA) Advisory Board Meeting hosted by Shire, October 2018. BZ reported paid lectures for Flynn Pharma. MAr received sponsorships to educational/scientific meetings and honoraria for consultancy and educational talks from Janssen, Lily, Takeda (Shire), and Flynn-pharma. SCo declares reimbursement for travel/accommodation expenses and honoraria in relation to lectures/courses delivered for the Association for Child and Adolescent Health (ACAMH), Canadian ADHD Alliance Resource (CADDRA), British Association of Psychopharmacology (BAP), and Healthcare Convention. PA/King's College London (KCL) received honoraria for consultancy to Takeda/Shire, Eli-Lilly, Medice, Novartis and Janssen, and for speaking at sponsored events for Shire, Lilly, Flynn Pharma, Medice, Novartis and Janssen. KCL was supported by funds for education and research from Shire, Medice, Flynn, Janssen, Vifor Pharma, GW Pharma, and QbTech. The remaining authors declare that the research was conducted in the absence of any commercial or financial relationships that could be construed as a potential conflict of interest.

## Abbreviations

ACAS, advisory: conciliation and arbitration service
ADHD: attention-deficit/hyperactivity disorder
AMHS: adult mental health services
ASD: autism spectrum disorders
CAMHS: child and adolescent mental health services
CBT: cognitive behavioral therapy
CCH: community child health
CCG: clinical commissioning group
DSM: diagnostic and statistical manual of mental disorders
FOI: freedom of information
GP: general practitioner
GPwERs: GPs with extended roles
IAPT: improving access to psychological therapies
ICD: international classification of disease
NHS: national health service
NICE: national institute for health and care excellence
UK: United Kingdom
UKAP: UK ADHD partnership
UKAAN: UK adult ADHD Network
SIGN: Scottish intercollegiate guidelines network.
